# Fossoriality in desert-adapted tenebrionid (Coleoptera) larvae

**DOI:** 10.1038/s41598-022-17581-6

**Published:** 2022-08-02

**Authors:** Marcin Raś, Marcin Jan Kamiński, Dariusz Iwan

**Affiliations:** 1 Zoological Museum, Museum and Institute of Zoology, Polish Academy of Sciences, Wilcza 64, 00-679 Warszawa, Poland; 2 Department of Entomology, Purdue University, 901 W. State Street, West Lafayette, IN 47907 USA

**Keywords:** Entomology, Behavioural ecology, Animal behaviour

## Abstract

In many extreme arid ecosystems, insects constitute major faunal components and are key contributors in nutrient cycling. Previous research on xerophily in insects has focused on adult forms. This study investigates skeletomuscular and behavioural adaptations of the Kalahari sandworm beetle larvae (*Gonopus tibialis* Fabricius) for dwelling in the sand. Microcomputed tomography enabled cuticle thickness distribution analysis, revealing structural reinforcements of the mandibular edge, the middle part of the head, and the ventral side of the front legs. Laboratory observations and the analysis of muscular system allowed for the definition and functional description of the elements of the digging apparatus of the sandworm larvae. Obtained results point to the crucial role of the head and mandibles in the digging process. These observations are important for understanding desert ecology and pose a challenge to develop newer excavation techniques.

## Introduction

With over 30,000 species darkling beetles (Tenebrionidae Latreille) are one of the most taxonomically diverse beetle families^1–3^. Although they can be found in a variety of different habitats (e.g. steppes, temperate and rain forests, ants/termite nets), the family is primarily known for a xerophilous lifestyle of many of its representatives^4^. In several extremely arid ecosystems, such as the Atacama, Namib, or Kalahari deserts, darkling beetles constitute a major component of the arthropod fauna and are a key element in nutrient cycling^1,5–10^. The extreme xerophily of tenebrionids has fascinated researchers from a wide variety of fields. As a result species such as the Namibian fog basking beetle (*Onymacris unguicularis* Haag) have been incorporated into biology-inspired engineering projects seeking solutions for efficient cooling and hydration systems^11,12^. Nevertheless, available studies on this matter are often taxonomically restricted leaving the dazzling potential of darkling beetles largely untouched—especially in the context of larval behaviour and ecology.

Considering the entire taxonomic diversity of Tenebrionidae, the majority of species probably have detritivorous, soil-dwelling larvae^1^. Particular adaptations to this lifestyle have never been closely studied. However, some morphological convergences were reported between relatively distant psammophilous phylogenetic groups^13–15^. For instance, the larvae of the subfamily Blaptinae Leach and some tribes within Pimeliinae Latreille are characterized by enlarged front legs^16–18^. Although representatives of those groups are often sympatric in arid and sandy habitats^19^, available molecular data suggest these lineages diverged over 150 Ma^20^. Except for the enlargement of prolegs, which presumably are adapted for digging in the sand^13^, not much is known about the strategies used by the larvae of different tenebrionid species to thrive in desert ecosystems. This concerns basic issues such as the movement style, not to mention specific skeletal and muscular adaptations.

The lack of studies on the larval adaptations in darkling beetles is mainly caused by the scarcity of available larval descriptions in general. The morphology of immature stages is known only for 2–8% of the described species, while only a fraction of these contributions provide any type of data on the lifestyle of the studied taxa^21,22^. In this context, the sandworm beetle [*Gonopus tibialis* (Fabricius)] and the subsocial desert beetle [*Parastizopus armaticeps* (Péringuey)] are exceptional, as their biology is relatively well understood^23–26^. Additionally, both species occur sympatrically on the sand dunes of the Kalahari Desert^27,28^. The adult forms in both cases are nocturnal fossorial detritivores that shelter in burrows during the daytime^24^. Although the lifestyle of the imaginal forms seems to be highly convergent, both taxa use different larval strategies. The larvae of the sandworm beetle simply dwell in the sand in search of food and suitable conditions, while those of the subsocial desert beetle inhabit burrows made by adults, which also feed and care for them^29–31^. Preimaginal stages of *P. armaticeps* do not dig or construct burrows themselves; however, they can move from burrow to burrow by following the adults. Furthermore, unlike the sandworm beetle, the pupating larvae of this species spin silk cocoons directly within the burrows^30,32^.

Because both above-mentioned species are sympatric, represent a single tenebrionid subfamily (Blaptinae), and their phylogenetic position is well established^15^, they constitute an interesting model for investigating larval adaptations to thrive in desert ecosystems. This study employs a variety of methods, including microcomputed tomography (micro-CT) to investigate the skeletomuscular adaptations of the sandworm beetle larvae for dwelling in the sand.

## Materials and methods

### Insects

This study focuses on analyzing the larvae of the sandworm beetle (*Gonopus tibialis*). However, two other tenebrionid species, subsocial desert beetle (*Parastizopus armaticeps*) and mealworm beetle (*Tenebrio molitor* L.) were used for reference. Both, *G. tibialis* and *P. armaticeps* belong to the same subfamily of darkling beetles (Blaptinae) representing two closely related tribes—Platynotini Mulsant & Rey and Opatrini Brullé respectively^15^. Unlike the preceding species, the mealworm beetle is classified within Tenebrioninae^3^. This species is a commonly used model organism for various types of biological studies^33–39^. In nature, mealworms inhabit decaying wood of different deciduous tree species in the temperate zone where they forage and feed on fungi, frass, and dead arthropods^40^.

The larvae of sandworm and subsocial beetles were obtained in rearing experiments from adults collected at the Kuzikus Wildlife Reserve in Namibia (coll. permit number 1738/2012), while individuals of *T. molitor* were commercially purchased. All investigations were based on the last larval instar. Detailed morphological descriptions of the larval stages of the Kalahari and subsocial beetles were published by Schulze^29,41^. Morphological nomenclature for skeletal structures follows that of Lawrence et al.^42,43^ and Beutel & Friedrich^44^. Muscular terminology was adopted after Fredrich and Beutel^45^, Beutel et al.^46^, and Aibecova et al.^47^. Images of selected morphological features were taken with a Hitachi S-3400N SEM.

### Observation of burrowing

**Figure 1:**
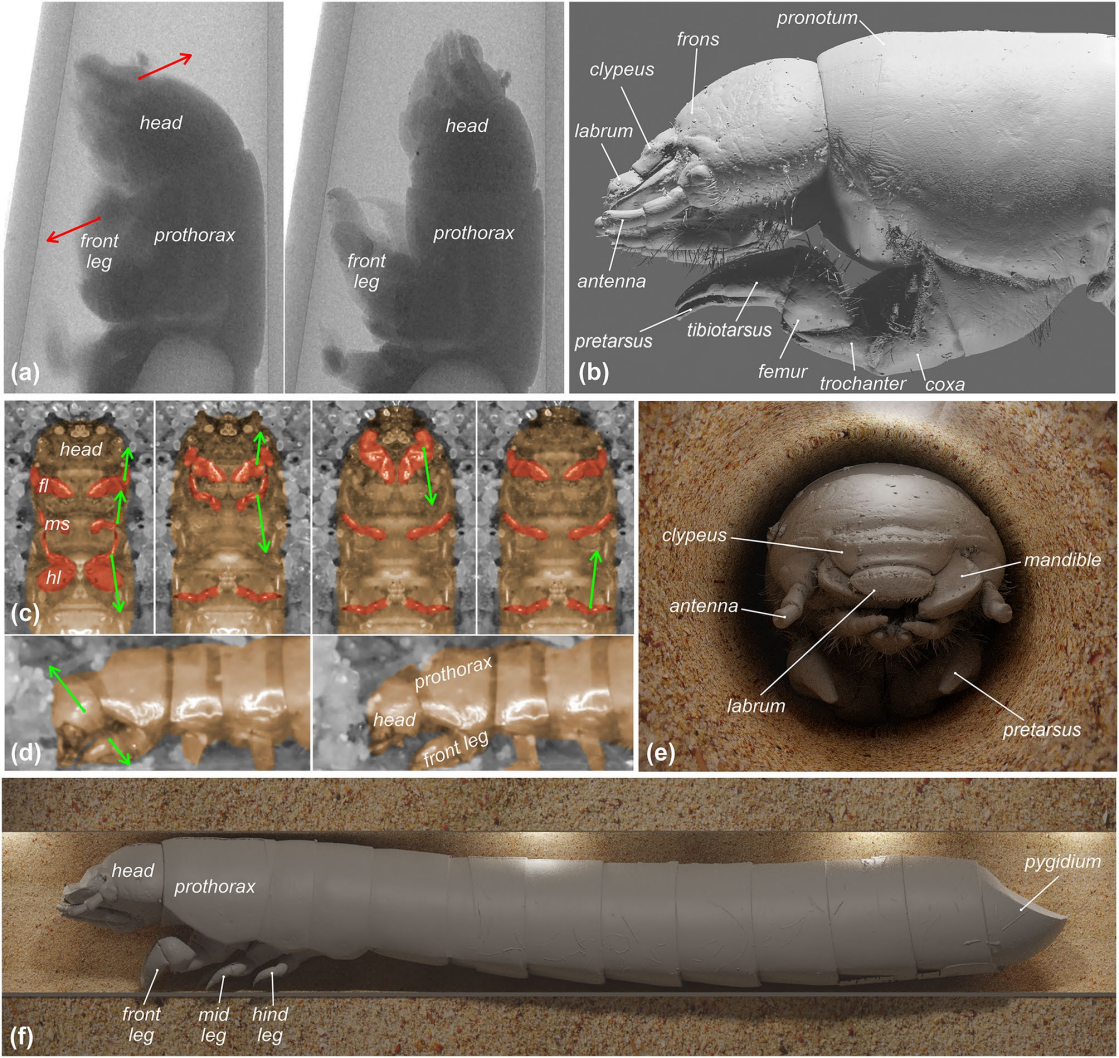
Burrowing dynamics and morphology of the sandworm beetle (*Gonopus tibialis*) larva. (**a**) Burrowing images obtained with microCT highlighting changes in head and front legs position. (**b**) Lateral view of larva imaging the relative position between head and front legs. (**c**) Ventral and (**d**) lateral burrowing images (fl—front legs, ml—middle legs, hl—hind legs). (**e**) frontal and (**f**) lateral microCT-based visualizations of digging larva. Arrows indicate the motion of particular elements.

Films depicting head and leg movements of sandworm beetle larvae were made using a SkyScan 1172 micro-CT system (see below) and a digital camera (Canon EOS D6 mark II with Tamron 90 mm lens). For movement analysis using the micro-CT, larvae were placed in pipette tips and a series of images were made. Subsequently, photos were assembled into a movie (Fig. 1a, Supplement 1^48^). The tip of the pipette simulated the passage in which the larvae move. For movement analysis carried out using a digital camera, larvae were placed on a Petri dish with sand on it. The camera was installed beneath this stage. The resulting movies display the movement of larvae beneath the sand (Fig. 1c,d, Supplement 2^48^). All experiments were conducted at room temperature, without specific humidity modifications. Altogether, over 45 min of footage for 13 sandworm beetles were recorded and analysed. The same experiments were conducted with mealworms in order to verify if digging strategies of different tenebrionid larvae are convergent. As larvae of the subsocial beetle do not dig directly in the soil they were not subjected to this type of experiment.

### Micro-CT analysis

**Figure 2:**
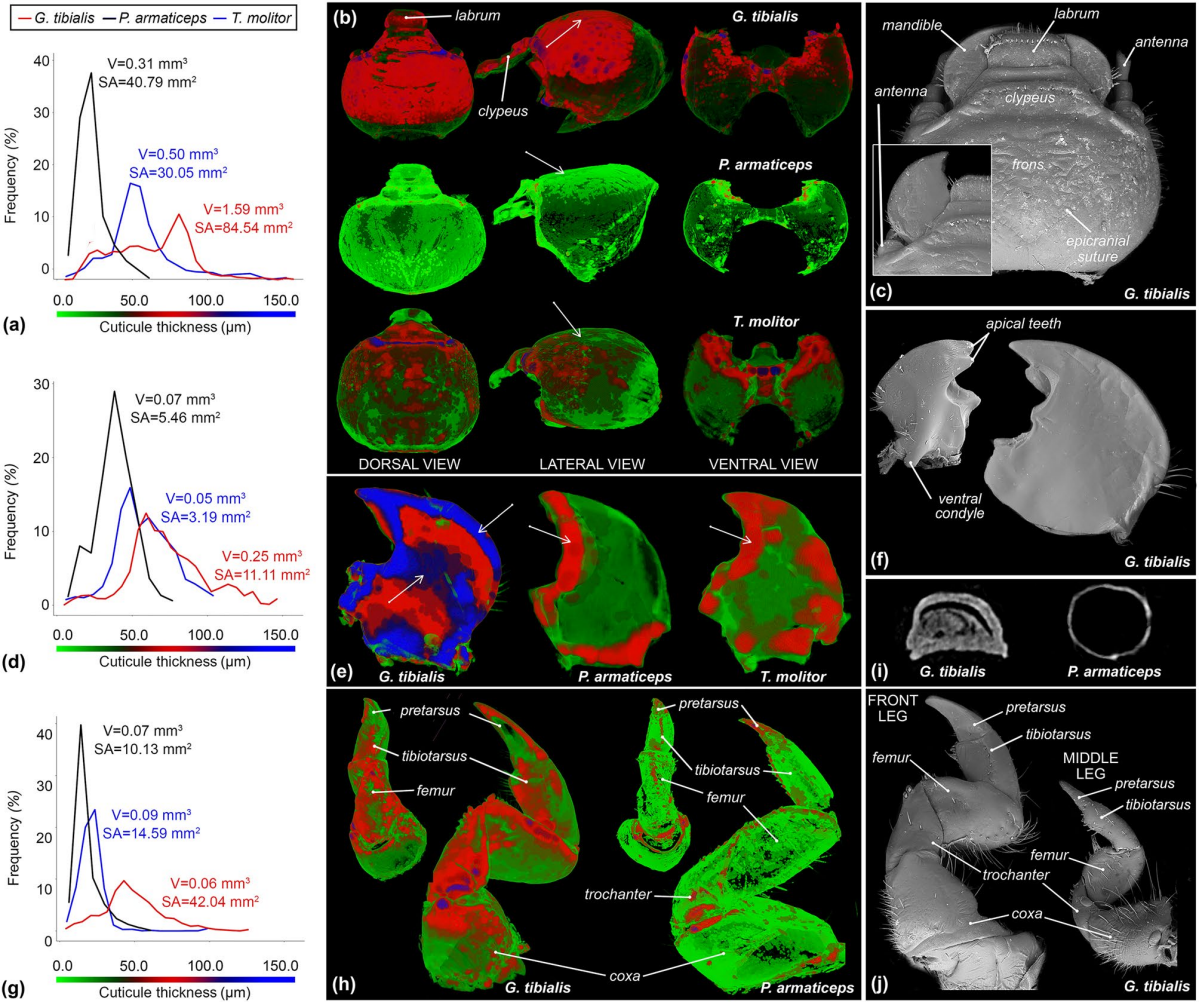
Cuticle thickness analysis dynamics and detailed morphology of larvae of the sandworm beetle (*Gonopus tibialis*), subsocial beetle (*Parastizopus armaticeps*), and mealworm (*Tenebrio molitor*). (**a**) cuticle thickness distribution of heads, with the exclusion of appendices (i.e. mandibles, antennae, maxillary palpi). (**b**) MicroCT-based visualizations of cuticle thickness distribution between heads of studied beetle species. (**c**) head morphology of the sandworm beetle larva. (**d**) cuticle thickness distribution of rights mandibles. (**e**) Visualizations of cuticle thickness distribution between right mandibles. (**f**) mandibular morphology of the sandworm beetle larva. (**g**) cuticle thickness distribution of front legs (right body side). Arrows indicate major differences between studied species. (**h**) Visualizations of cuticle thickness distribution between front legs. (**i**) transverse digital cross sections of front tarsungulus (**j**) leg morphology of the sandworm beetle larva.

Analyses were performed using a SkyScan 1172 system^49^. Before scanning specimens underwent the following preparation process: fixation in 4% buffered formaldehyde with 2% glutaraldehyde, desiccation through 30, 50, 70, and 96% ethanol (each for about one day). Subsequently studied samples were dried in a critical point dryer. All specimens were scanned with the lamp set to voltage 40 kV and current to 250 μA with matrix resolution set for 4000 × 4000 pixels. As a result, the following pixel sizes were obtained: 2.71 μm for the sandworm beetle, 3.86 μm for the subsocial beetle, and 3.06 μm for the mealworm. Segmentation was performed in CTAn Brucker (version 1.18.4.0 +), while visualizations and renders in Blender (version 3.0.0). Analysis of cuticle thickness was carried out in the CTAn. For this analysis, all appendages of the head (e.g., antennae, maxillary palpi) were digitally removed (Fig. 2b). All raw micro-CT datasets are accessible from the Harvard Dataverse repository (Supplement 3–5^48^). As no substantial differences in morphology were observed between the legs and mandibles of both body sides, measurements (volume, thickness) were taken from the right side of the studied specimens.

### Statistical analysis

All statistical analyses and charts were made in the R environment (version 4.0.2^50^). To evaluate the differences in cuticle thickness distribution between the studied species, obtained data were normalized by using the values width of the head capsule (Supplement 6^48^). Differences were evaluated with a non-parametric, two-sample Kolmogorov–Smirnov test procedure^51^. P-value of less than 0.05 to be a statistical significance.

## Results

### Digging movements

When tunnelling straight, the larval legs of particular thoracic segments are moving symmetrically. The forward motion of the larva is initiated by the hind legs, which are moved back from the level of the second thoracic segment all the way to the base of the third. During this phase, the head is retracted, while its dorsal surface pushes against the sand. The first pair of legs is hidden underneath the head, pointing anteriorly (Fig. 1c). While the legs of the third pair are moved backward, the second pair is moving forward. Their contact with sand, and the beginning of reverse motion, are initiated once the third pair of legs reaches its maximum extension. Similarly, once the midlegs reach their maximum amplitude, the front legs are tilted, providing support for the straightening of the head, which is the main digging element (Fig. 1a). Larval tunnels are mainly formed by head movements. In particular, the dorsal surface is compressing the sand in the upper part of the tunnel profile (Fig. 1d). Conducted observations suggest that legs are not directly involved in digging or even moving the sand backward, but rather drive the forward motion of the larva (middle and hindlegs) and actively support the digging motion of the head (front legs). When fully extended forwards, the front legs are not exceeding beyond the ventral outline of the head (Fig. 1b). Furthermore, the presence of the abdomen inhibits any direct involvement of the legs in the sand removal process (Fig. 1e,f). When exposed to the experimental conditions, mealworms displayed fully convergent digging behaviours.

### Digging apparatus

Observations described above resulted in the identification of the basic elements of the digging apparatus of the sandworm beetle larva. The morphological specification of these elements is provided below.

#### Head

Sandworms are characterized by the absence of eyes (Fig. 1b). Within the set of investigated species, the lack of ocelli has also been reported in the case of the subsocial desert beetle. Eyes are clearly detectable in the case of the mealworm beetle. When compared to reference taxa the sandworm beetle is the only species in which the head capsule is subequal to the pronotum in width (head\pronotum width ratio: *G. tibilis* = 0.98–0.99, *P. armaticeps* = 0.66–0.77, *T. molitor* = 0.78–0.82). Furthermore, the mandibles of this species are uniquely expanded laterally creating a relatively large surface that can shield the antenne dorsally once the mandibles are opened (Fig. 2c). The cuticle of mandibles in the scanned individual of the sandworm beetle has a surface of 11.11 mm^2^ and a volume of 0.25 mm^3^ (ratio = 44.4). Corresponding values measured for the subsocial desert beetle are as follows: 5.46 mm^2^ and 0.07 mm^3^ (ratio = 75.6). Finally, for the mealworm beetle: 3.19mm^2^, 0.05 mm^3^ (ratio = 56.9).

#### Legs

The prothoracic legs of the sandworm beetle species are noticeably enlarged (Fig. 2j). The combined length of pretarsus and tibiotarsus is over two times greater than that of the corresponding morphological elements of hind legs (Fig. 2j). Contrarily, in the case of the employed reference species, the front legs are only slightly enlarged (length ratio of fore/hind pretarsus + tibiotarsus = 1.4–1.5). The sandworm beetle is the only one within the studied species to possess tuberculation on the front legs (Fig. 2j). Moreover, the propretarsus of this species is uniquely flattened (Fig. 2i,j). The cuticle of the forelegs in the scanned individual of the sandworm beetle has a surface of 42.04 mm^2^ and a volume of 0.62 mm^3^ (ratio = 67.81). Corresponding values measured for the subsocial desert beetle are as follows: 14.59 mm^2^ and 0.09 mm^3^ (ratio = 162.1). Finally, for the mealworm beetle: 10.13 mm^2^, 0.07 mm^3^ (ratio = 144.7). The propretarsus in the sandworm beetle is about as long as the protibiotarsus (Fig. 2h,j). It is convex dorsally, while ventrally flat, and possesses a rounded apex (Fig. 2i). In contrast, the propretarsus of the subsocial beetle is almost two times shorter than the tibiotarsus. It has a pointed tip, while the ventral surface is convex. SimilarlyInthe protibiotarsus of the mealworm is almost twice as long as the propretarsus.

### Thickness analysis

The following section presents the results of the cuticle thickness analysis conducted for the selected elements of the digging apparatus of the sandworm.

#### Head

The cuticle of the dorsal side of the head capsule of the sandworm beetle is noticeably thicker than that of the two reference species (Fig. 2a,b). However, it should be noted that the larvae of this species are on average larger than those of the other species. Nevertheless, the thickness distribution in the case of the sandworm beetle noticeably shifted towards the higher values (Fig. 2b). This difference was statistically significant (*G. tibialis*—*P. armaticeps*: D = 0.40, P < 0.01; *G. tibialis*—*T. molitor*: D = 0.25, P < 0.001). In the case of that species, the most frequent value (mode) is 81.0 ± 3.0 μm (12.73%), while in the mealworm 49.0 ± 3.0 μm (19.0%), and finally in the subsocial desert beetle 23.0 ± 4.0 μm (39.78%). Recorded mean values of cuticle thickness for these species were 81.0 μm, 79.0 μm, and 35.0 μm respectively. Cuticle with thickness below mode values constitutes around 67.7% of the total volume of the head capsule in the sandworm beetle, 29.5% in mealworm, and 35.8% in the subsocial beetle.

The thickest element (over 120.0 μm) occurring on the head capsule of the sandworm beetle is the frontoclypeal suture and its extension along the front edge of the gena over the antennal insertions (Fig. 2b). The thickness of the frons and dorsal parts of the gene is also relatively high and ranges between 50.0 to 100.0 μm. A thick cuticle is also present in the lateral margins and base of the clypeus (75.9 μm), lateral edges of the labrum (65.1 μm), and the rod running across its entire width at its center (65.1 μm). The thickness of the ventral side of the head of sandworm beetles did not exceed 50.0 μm. The only thickened elements form a ridge on the border of the gulamentum (Fig. 2b). The thickening of the gulamentum forms the base for the posterior tentorial arms.

Imaging of the cuticle thickness distribution revealed the subsocial beetle differs mainly by the lack of reinforcement of the mid part of the ventral side of the head (Fig. 2b). Thickened elements (> 20.0 μm) of this side of the head occur around the antennal insertions and run along the frontoclypeal suture (Fig. 2b). The clypeus itself is only slightly thicker and the labrum is built from a cuticle of a relatively low thickness (< 20.0 μm). On the ventral part of the head capsule, the cuticle is reinforced in the maxillary fossa (Fig. 2b). Similarly, the mid part of the ventral side of the head of the mealworm beetle is also not reinforced (Fig. 2b), while the differences in cuticle thickness between the ventral and dorsal sides of the capsule are less highlighted than in the case of the sandworm beetle. In general, cuticle thickness distribution for ventral sides of the head are highly convergent among all studied species (Fig. 2b).

#### Mandibles

Thickness distribution is similar in all studied species, and the highest percentage of cuticle has an intermediate thickness (Fig. 2d,e). The most abundant chitin in the sandworm beetle (12.04%) has a thickness of 59.0 ± 3.0 μm, while in the mealworm (15.84%) 49.0 ± 3.0 μm, and 38.0 ± 4.0 μm (28.96%) in the case of the subsocial desert beetle.

In the sandworm beetle the anterior and external mandible edge, apical teeth, molar lobe, articulatory condyles, and posterior edge of mola are the thickest elements (> 100.0 μm). The reinforcement of the outer mandibular edge is characteristic for this species when compared to the referencs (Fig. 2e). On the other hand, it can be concluded that the thickening of the inner mandibular edge is common for all of the studied species.

#### Front legs

Thickness distribution charts in the case of the subsocial desert beetle and mealworm are skewed towards lower values (Fig. 2g,h). A relatively thin cuticle (11.0–19.0 μm and 21.0–27.0 μm respectively) represents 60.80% of the total volume of the cuticle in the subsocial beetle and 31.91% in the mealworm. In the case of the sandworm beetle, the most abundant chitin (14.90%) has a thickness of 43.0 ± 3.0 μm. In this species, the thickest noted element has 124.0 ± 3.0 μm, compared to 97.0 ± 3.0 μm in the mealworm, and 61.0 ± 4.0 μm in the subsocial beetle.

The thickest cuticle (> 100.0 μm) in the case of the sandworm beetle occurs in the ventral and anterior parts of the coxa, trochanter, and femur. The thickening of the cuticle can also be observed on the ventral edge of the pretarsus. In both, the subsocial beetle and mealworm only the apex of the pretarsungulus and minor parts around the coxal base and coxo-trochanteral articulation possess thickened cuticle (Fig. 2h).

### Musculature

The following section describes the musculature of the digging apparatus of the sandworms.

#### Head

**Figure 3:**
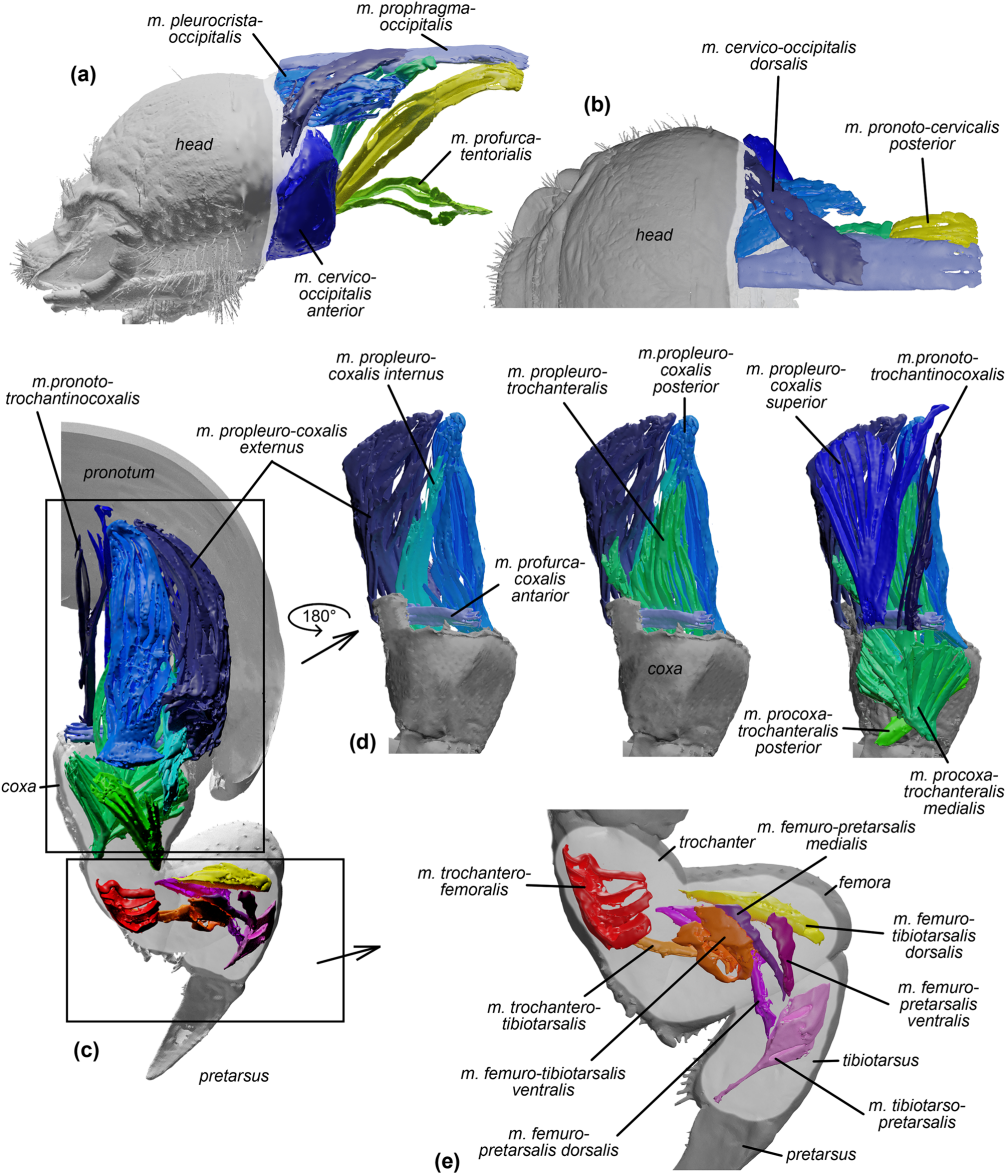
The musculature of the digging apparatus of the sandworm beetle (*Gonopus tibialis*) larva. (**a**–**c**) head: (**a**) lateral and (**b**) dorsal views. (**c**–**e**) front leg: (**c**) posterior, (**d**) frontal, (**e**) lateral views. Detailed descriptions of particular muscles are presented in Table 1.

The head movements are operated by seven double muscles (Table 1). Three of them enable the uplift of the head, and with a total volume of 0.43 mm^3^, they constitute the largest muscle group involved in head movements (Table 1, Fig. 3a,b). The three antagonistic muscles have a total volume of 0.18 mm^3^. Lateral movements of the head are carried out by a single muscle, which has a volume of 0.17 mm^3^ (Table 1).

**Table 1:** The musculature of the digging apparatus of the sandworm beetle (*Gonopus tibialis*) larva.

Location	Muscle	Insertion/Origin	Volume
Head	m. cervico-occipitalis anterior *	Dorslolateral part of occipita/Latero-anterior part of basisternum anterior flange	0.24
	m. prophragma- occipitalis *	Dorsal area of occipita/Middle region of prophragma	0.07
	m. pleurocrista-occipitalis *	Pleural ridge anterad/Lateral area of occipitae	0.12
	*m. pronoto-cervicalis posterior*	Lateral region of first lateral cervical sclerite/Posterior half part of pronotum	0.09
	*m. pronoto-cervicalis medialis*	Central area of first lateral cervical sclerite/Central region of pronotum	0.05
	*m. profurca-tentorialis*	Posterior tentorial arms/Profurca	0.05
	m. cervico-occipitalis dorsalis **	Dorsolateral area of occipita/Anterior marigin of pronotum	0.17
Mandibles	*m. craniomandibularis internus*	Adductor apodeme/Almost whole lateral, dorsal and posterior walls of head	0.69
	*m. craniomandibularis externus*	Abductor apodeme/Lateral side of head	0.08
Legs/coxa	*m. propleuro-coxalis superior*	Anterior procoxal rim/Anterior area of propleuron	0.06
	*m. pronoto-trochantinocoxalis*	Anterior procoxal rim/Anteromedial part of pronotum	0.02
	*m. propleuro-coxalis externus*	Proximal posterior margin of coxa/Posterior wall of propleuron	0.21
	*m. propleuro-coxalis posterior*	Proximal posterior margin of coxa/Posterior wall of propleuron	0.13
	*m. propleuro-coxalis internus*	Proximal posterior margin of coxa/Posterior wall of propleuron	0.04
	*m. profurca-coxalis anterior *	Anterior procoxal rim/Lateral face of profurcal stem	0.01
*trochanter*	*m. procoxa-trochanteralis posterior*	Lateral proximal margin of trochanter/Posterior wall of coxa	0.03
	*m. procoxa-trochanteralis medialis*	Medial proximal margin of trochanter/Anterior, medial and lateral walls of proximal part of coxa, endopleura	0.15
	*m. propleuro-trochanteralis*	Trochanter/Propleural apodeme	
*femur*	*m. trochantero-femoralis*	Femur lateral margin/Medial wall of trochanter	0.02
*tibiotarsus*	*m. trochantero-tibiotarsalis*	Tibiotarsus medial proximal margin/Medial wall of trochanter	0.01
	*m. femuro-tibiotarsalis ventralis*	Tibiotarsus medial proximal margin/Ventral wall of femur	0.01
	*m. femuro-tibiotarsalis dorsalis*	Tibiotarsus ventrolateral margin/Dorsal wall of femur	0.01
*pretarsus*	*m. femuro-pretarsalis dorsalis*	Pretarsus tendon/Dorsal wall of femur	0.01
	*m. tibiotarso-pretarsalis*	Pretarsus tendon/Posterioventral wall of tibiotarsus	0.01
	*m. femuro-pretarsalis ventralis*	Pretarsus tendon/Anteroventral wall of femur	0.01
	*m. femuro-pretarsalis medialis*	Pretarsus tendon/Medioventral wall of femur	0.01

*Muscles uplifting the head.

**Muscles responsible for lateral movements.

#### Mandibles

Mandibular movements are operated by a pair of antagonistic muscles (Table 1). With a volume of 0.69 mm^3^, the adductor (*m. craniomandibularis internus*) constitutes the single largest muscle occurring within the head. The abductor (*m. craniomandibularis externus*) is much smaller and has a volume of 0.08 mm^3^.

#### Front legs

Altogether, 17 muscles were identified within the front leg of the sandworm beetle larva (Table 1, Fig. 3c–e). With a volume of 0.47 mm^3^, the muscles moving the coxa constitute the largest group, which is followed by the muscles of the trochanter (0.17 mm^3^). The muscles of the other leg segments have noticeably lower volumes, i.e. femur—0.02 mm^3^, tibiotarsus—0.03 mm^3^, and pretarsus—0.04 mm^3^.

## Discussion

Previously, several studies have investigated physiological, behavioral, and anatomical adaptations of different insect groups to arid environments. Nevertheless, the majority of these contributions concerned the adult epigeic forms. In this context, the most commonly mentioned adaptations of desert inhabiting beetles are the specific structuring of the elytra^52–55^, the phenomenon of fog basking^11^, the longevity of the adult forms^7,56,57^, the morphological modifications of the legs to the psammophilous way of life^13^, the reduction in egg production^58,59^, and modifications of circadian rhythms^60–62^. Data on larval adaptations are scarce and extremely general in their nature. It has been mentioned that larvae of some of the desert inhabiting tenebrionids bury themselves in the sand to avoid overheating^52^, and in the case of some species a rapid larval development following the rain season has been observed^63^. The scarcity of available information in this context is surprising as larval traits can determine the fitness of adult forms^64–66^, and therefore constitutes an important element for the functioning of a given ecosystem.

Our data revealed that the head is the main digging element, while the enlarged front legs constitute support for its up and down movements (Fig. 1). Analysis of chitin thickness distribution revealed key differences between the sandworm beetle and two reference taxa. Namely, the sand-dwelling larva of *G. tibialis* possesses unique sclerotizations of the outer edge of the mandibles and the center of frons (Fig. 2b,e). Furthermore, the dorsal surface of mandibles is flattened and extended sideways, providing a larger digging surface (Fig. 2f). The crucial role of the head in the digging process is also highlighted by the musculature, as the group of muscles responsible for the up motion of the head is significantly larger than the antagonistic one (Table 1). Taking into consideration data obtained during the observations it can be concluded that modifications of the front legs of sandworm beetle larva (presence of tubercles, reinforcement of coxal and trochanter chitin, flat pretarsus; Fig. 2) enable them to more firmly move through the sand.

Surprisingly, when buried in the sand, mealworms displayed identical digging behavior to the larvae of the sandworm beetle. This observation suggests that such a digging strategy might be omnipresent within the family of darkling beetles. Tenebrionid larvae—sometimes referred to as the false wireworms—are prone to homoplasy in their morphology^22,67^. Therefore, this generalistic digging strategy might enable different tenebrionid species to thrive in a wide variety of habitats, such as decaying wood, soil, or sand^1^. The majority of diagnostic features used in false wireworm descriptions concern the mouthparts and the pygydium^21,67–71^. Aside from the afore mentioned enlargement of the front legs no apparent modifications to the psammophilous lifestyle were ever listed.

## Conclusion

Our study shows that μCT-based thickness distribution analysis is a reliable tool for investigating particular adaptation problems within different insect groups. Specifically, our data revealed the following unique features of sand-digging tenebrionid larvae: thickening and extension of the dorsal surface of mandibles and extention of the inner side of protarsungulus, cuticle reinforcements on the outer mandibular edge, the middle part of the head, and the ventral side of front legs. Our observations also highlight the essential role of the head capsule and mandibles in the digging process.

## Data Availability

All supplementary data are available at Harvard Dataverse (https://doi.org/10.7910/DVN/NNAETE).
